# Embosphere microsphere embolization of the middle meningeal artery for the treatment of chronic subdural hematoma: a single-center experience

**DOI:** 10.3389/fneur.2025.1656184

**Published:** 2025-10-07

**Authors:** Ligang Xu, Dewei Zhang, Yeqing Jiang, Lei Zhang, Jinyan Xu, Weiyi Zhu, Wei Chen, Jun Guo, Xin Gu, Jun Wan

**Affiliations:** ^1^Department of Interventional Radiology, Jing’an District Central Hospital, Fudan University, Shanghai, China; ^2^Department of Neurosurgery, Jing’an District Central Hospital, Fudan University, Shanghai, China; ^3^Department of Radiology, Huashan Hospital Affiliated to Fudan University, Shanghai, China

**Keywords:** chronic subdural hematoma, hemorrhagic stroke, middle meningeal artery embolization, Embosphere, outcomes

## Abstract

**Background:**

To explore the efficacy, safety, and surgical experience of Embosphere microsphere embolization of the middle meningeal artery (MMAE) in the treatment of chronic subdural hematoma.

**Methods:**

A total of 25 patients with chronic subdural hematoma (4 cases with initial treatment, 5 cases with prophylaxis after burr hole drainage, and 16 cases with recurrence after surgical drainage) were selected from the Interventional Therapy Department of Shanghai Jing'an District Central Hospital from October 2022 to December 2024. The patient was treated with bilateral middle meningeal artery embolization under local anesthesia. The recurrence rate of postoperative hematoma and the proportion of patients with maximum hematoma thickness reduction > 50% at 3 months after intervention were used as the main indicators to evaluate efficacy. The complications and operation-related adverse events were observed, and the experience of interventional therapy was retrospectively summarized.

**Results:**

All 25 patients completed the interventional procedure. Among them, 1 patient underwent unilateral embolization due to the absence of the middle meningeal artery on the affected side, while 24 patients received bilateral embolization. A total of 45 middle meningeal arteries were embolized. After treatment, hematoma absorption and symptoms were improved in all patients, and no recurrence of hematoma was found. The treatment success rate was 100%. The 25 patients were followed up 3 months after the interventional procedure, and the proportion of patients with maximum thickness reduction of hematoma >50% reached 96%. There were no deaths and 2 asymptomatic complications, including 1 hemorrhage complication and 1 ischemia complication. Complications were not related to embolization materials.

**Conclusion:**

Embolization of the middle meningeal artery using Embosphere microspheres is safe and effective in the treatment of chronic subdural hematoma. For patients with recurrence or high risk of recurrence, bilateral middle meningeal artery embolization should be considered.

## Introduction

Chronic subdural hematoma (CSDH) is a common subtype of stroke in neurosurgery, characterized by high incidence and recurrence rates. With the global aging population and the widespread use of antiplatelet and anticoagulant medications, the incidence of CSDH has shown a significant upward trend (1). It is reported that by 2030, the number of CSDH patients will increase to 121.4 per 100,000, making it the most common disease in neurosurgery (2).

Craniotomy and drainage are the first-line treatments for CSDH, but the recurrence rate can be as high as 30% (3). The inflammatory response in CSDH produces vascularized membranes, which can prevent absorption and contribute to recurrence, with the blood supply to the membrane coming from the middle meningeal artery (MMA) (4). Middle meningeal artery embolization (MMAE) has been proven an effective adjunct therapy for surgical and non-surgical CSDH treatments (5, 6). Currently, the main materials used for middle meningeal artery embolization are Onyx, emboparticles polyvinyl alcohol (PVA), and n-butyl cyanoacrylate (n-BCA), and coils, with Onyx being the most widely used (7). Compared to these materials, the traditional embolic agent Embosphere microspheres are simple to operate, have a short surgical duration, and are cost-effective. However, the conclusions obtained from the limited literature reports on Embosphere microspheres are completely opposite (8, 9). Therefore, this center conducted a retrospective analysis of the efficacy and safety of Embosphere microspheres in treating 25 cases of CSDH.

## Methods

All patients or their legal representatives signed the informed consent for minimally invasive interventional surgery. The study was approved by the Ethics Committee of Shanghai Jing'an District Central Hospital (Batch number: 2023-27). The data of 32 consecutive patients with CSDH between September 2022 and December 2024 were sourced from the prospective case database of the Interventional Therapy Department at Jing'an District Central Hospital in Shanghai. Seven cases were excluded due to combined Onyx embolization. Finally, 25 cases were included in this study.

### Inclusion criteria

- Age ≥ 18 years.
- Diagnosis of CSDH confirmed by imaging studies (head CT and/or MRI).
- Presence of neurological symptoms related to the mass effect of the hematoma (e.g., headache, dizziness, muscle weakness, seizures, decreased consciousness, etc.).
- Treated by MMAE.

### Exclusion criteria

- CSDH with the maximum thickness in the axial slice of hematoma < 10 mm in crainal CT.
- CSDH with underlying causes, such as concomitant cerebrovascular diseases, brain tumors, arachnoid cysts, etc.
- Previous MMAE for other vascular diseases.
- Refusal of surgical or interventional procedures.
- Patients with severe heart, liver, or kidney failure, cachexia, or an expected survival period of less than 6 months.
- Inability to adhere to follow-up schedules or poor patient cooperation.

### Endovascular treatment

The procedure was preferably under local anesthesia, while general anesthesia was chosen for elderly patients who may have difficulty cooperating during the Endovascular treatment. The 6F artery sheath (Terumo, Japan) was inserted by the femoral artery approach. Whole cerebrovascular angiography was performed with a 5\u00B0F Headhunter angiography catheter (Cordis, United States) to determine whether there were other cerebrovascular lesions and vascular anatomical variations, and to confirm the integrity of cerebrovascular before embolization. The 5/6F Envoy guiding catheter (Cordis, United States) was then replaced and placed in the external carotid artery. The Echelon 10 microcatheter (Ev3, United States) was superselectively inserted into the MMA trunk under the guidance of a 0.014-inch synchro2 microguide wire (Stryker, United States). Angiography via the microcatheter was performed to thoroughly assess the distal branches of the MMA (include the anterior and the posterior branches) for abnormal staining, contrast extravasation, or anastomosis with the ophthalmic artery. If no anastomosis with the ophthalmic artery is present, embolization can be directly performed on the main trunk distal to the Foramen spinosum, which not only reduces the risk of vascular rupture and bleeding but also saves surgical time. If there were any potentially hazardous anastomoses of the collateral vessels, further superselection of the microcatheter over the anastomotic branch would be required. The embolic agent used is diluted (contrast agent and saline mixed in a 6:4 ratio) Embosphere microspheres (Merit Medical, United States). Based on the clinical experience of this center, the 6:4 contrast-saline dilution ratio takes into account both the enhancement of fluoroscopic visibility and the efficiency of embolization. Particles with a diameter of 100–300 um are routinely used. When the distal branches have been embolized, larger particles of 300–500um can be used to embolize the more proximal vessels to shorten the procedure time. Before embolization with Embosphere microspheres, infuse 1 mL of fasudil to dilate blood vessels and prevent vasospasm, which facilitates the particles to drift with blood flow toward the peripheral microcirculation. The key point of embolization is to inject the embolic agent in a pulsed and slow manner. The injection pressure is changing in real time. Under the condition of the highest DSA radiation dose and the largest screen size, the injection pressure is adjusted to maintain forward blood flow without reflux. On one hand, this ensures dense embolization of the distal microcirculation, and on the other hand, it avoids reflux and accidental embolization. The endpoint of embolization is the stasis of contrast in the target vessel. A repeat angiography after 5 min showing no clear visualization of the anterior and posterior branches of the MMA indicates successful embolization. Finally, bilateral internal carotid artery angiography is performed to ensure no missing vascular branches. Due to potential anastomosis of bilateral MMA, symptomatic recurrent patients were embolized by bilateral MMA.

Our institutional experience with MMAE using Embosphere microspheres such as follows: 1. Contrast-Saline mixture preparation – A 6:4 ratio of contrast agent to saline is used to create a homogeneous suspension, enhancing fluoroscopic visibility during injection. 2. Embolization Techniques – (1) Distal Embolization: Slow injection of 100–300 μm Embosphere microspheres allows the particles to follow blood flow and diffuse into the terminal meningeal artery branches, achieving dense embolization of the distal microvasculature. Minimizes collateral compensation by occluding the terminal circulation. (2) Sequential Embolization: ① Perform initial distal embolization with 100–300 μm microspheres. ② Follow with 300–500 μm microspheres to occlude proximal branches and the main trunk. Ensures comprehensive embolization while reducing material usage and shortening procedure time. Balances efficacy and cost-effectiveness. If dangerous anastomotic branches are present, prioritize distal embolization to avoid inadvertent occlusion of critical collateral pathways. If no dangerous anastomoses are identified, sequential embolization is preferred for its efficiency and cost-saving benefits. Isolated main trunk embolization fails to achieve dense terminal occlusion, allowing rapid collateral recruitment from adjacent meningeal arteries and increasing recurrence risk. Key Technical Principles are slow and controlled injection to ensure optimal distal penetration of microspheres and prevent reflux-related ectopic embolization and vascular injury. Adjust injection speed based on flow dynamics to maintain distal embolization precision. 3. Routine full cerebral angiography: Before embolization, complete cerebral angiography (including bilateral internal carotid arteries, external carotid arteries, and vertebral arteries) is performed to comprehensively evaluate intracranial vascular pathologies (e.g., arteriovenous shunts, aneurysms) and identify anatomical variations (e.g., collateral pathways, dangerous anastomoses) and establish a baseline for pre- and post-embolization comparison to detect procedural complications (e.g., unintended branch occlusion). 4. Microcatheter positioning strategy: The microcatheter should bypass the openings of dangerous anastomotic branches, such as the recurrent branch of the ophthalmic artery, to prevent ectopic embolism and subsequent neurological dysfunction. Embospheres were injected in the MMA distal to the Foramen spinosum to prevent accidental embolization of its petrosal branch. The microcatheters were superselectively advanced into the anterior and posterior branches of the middle meningeal artery, respectively, with the catheter tip positioned distal to the orbital apex in the anterior branch whenever possible. Given that the posterior branch is relatively thin, the microcatheter was generally placed in the proximal one-third segment of the posterior branch. If the posterior branch originated distal to the foramen spinosum and there was an absence of ophthalmic artery anastomosis in the anterior branch, the microcatheter could be positioned in the main trunk distal to the foramen spinosum for embolization. 5. Before embolization with Embosphere microspheres, infuse 1 mL of fasudil to dilate blood vessels and prevent vasospasm, which facilitates the particles to drift with blood flow toward the peripheral microcirculation, especially suitable for slender petrosquamosal branches. 6. Bilateral MMA embolization for CSDH: For patients with unilateral chronic subdural hematoma, after embolization of the affected side, angiography of the contralateral middle meningeal artery should be performed. Anteroposterior projection angiography is conducted first, followed by lateral projection to ensure that the initial angiogram can visualize anastomotic branches crossing the midline. If contralateral middle meningeal artery branches with midline-crossing anastomoses are identified, superselective embolization of these branches should be performed. If no such anastomoses are observed, embolization may be omitted unless accompanied by high-risk factors (such as use of anticoagulant medications, coagulation abnormalities, or recurrent hematomas, etc.).

### Postoperative evaluation and follow-up

Postoperative follow-up cranial CT scans were conducted at 1 month and 3 months to evaluate the absorption of the hematoma. The primary assessment metrics included: the proportion of patients with a reduction in maximum hematoma thickness of >50% at 3 months, and the postoperative hematoma recurrence rate. Recurrence was defined as a persistent subdural hematoma on cranial CT, accompanied by ongoing or new symptoms related to CSDH. Secondary assessment metrics included: mortality rate, complication rate, and modified Rankin Scale score (mRS).

### Statistical methods

All data were analyzed by SPSS 25.0 software. The ‌‌Shapiro-Wilk test was used to the normalcy of a distribution. X̅ ± s represented the continuous variables that conform to the normal distribution, and those that do not conform to the normal distribution were described by M (median) and IQR (interquartile range). Categorical variables are expressed by frequency and proportion.

## Results

### Baseline characteristics

The patients had a mean age of 71 ± 10 years (range: 50–91 years), including 22 males and 3 females. Fourteen patients (56%) had a definite history of trauma. The main symptoms included headache (60%, 15 cases), dizziness (52%, 13 cases), and minor motor weakness (44%, 11 cases). Comorbidities included hypertension in 8 cases (32%) and diabetes mellitus in 5 cases (20%). One patient (4%) was on long-term anticoagulant therapy, and another (4%) received antiplatelet therapy. Bilateral hematomas were observed in 11 patients (44%), and unilateral hematomas in 14 patients (56%), totaling 36 hematoma sides. The maximum hematoma thickness on admission was 16.4 ± 6.5 mm. Treatment modalities included: primary MMAE in 4 cases (16%), adjunct embolization after burr hole drainage in 5 cases (20%), and salvage embolization following recurrence after burr hole drainage in 16 cases (64%) (Table 1). Among the patients who received adjunct embolization, 2 cases were performed 7 days after burr hole drainage, and 3 cases were carried out 5 days after burr hole drainage.

**Table 1:** Clinical and demographic details of study population.

Variable	Number (*n*%)/mean + SD
Male	22 (88%)
Age (years)	71 ± 10
Presenting symptoms	
Headache	15 (60%)
Dizziness	13 (52%)
Minor motor weakness	11 (44%)
Comorbidities	
Hypertension	8 (32%)
Diabetes mellitus	5 (20%)
Antiplatelet/anticoagulation medication	
Dual antiplatelet	1 (4%)
Anticoagulation	1 (4%)
SDH characteristic	
Bilateral side	11 (44%)
Maximum width (mm)*	16.4 (6.5%)
Treatment modalities	
Recurrence	16 (64%)
Adjunct treatment	5 (20%)
Primary treatment	4 (16%)
Bilateral MMAE	20 (80%)
MMA-ophthalmic artery anastomosis	3 (12%)
Embolization type (45 sides)	
Distal embolization	30 (66.7%)
Combined embolization	15 (33.3%)
Isolated main trunk embolization	0 (0%)

SD (standard deviation), SDH (subdural hematoma), mm (millimeters), MMAE (middle meningeal artery embolization).

### Intervention characteristics and follow-up evaluation

Bilateral MMAE was successfully performed in 20 patients, and affected side MMAE in 5 patients. Thereinto, contralateral MMAE was successfully performed in 1 patient due to the absence of MMA on the affected side. Among the patients with bilateral MMAE, 11 had bilateral hematomas and 9 had unilateral hematomas. Among unilateral hematomas patients with bilateral MMAE, eight were recurrent cases, showing cross-midline anastomosis of branches of the contralateral middle meningeal artery. The remaining 1 case showed no clear anastomosis. Bilateral MMAE was carried out because of a recurrent patient taking blood-activating medications. A total of 45 sides were embolized. Middle meningeal artery-ophthalmic artery anastomosis was observed in 3 patients. Distal embolization was performed in 30 sides, combined distal branch and main trunk embolization in 15 sides, and no patients underwent isolated main trunk embolization (Table 1).

All 25 patients with CSDH demonstrated significant symptomatic improvement after treatment, and no recurrence of hematoma was found. Among them, 25 patients underwent three-month post-procedural cranial CT follow-up, with 96% of patients showing a > 50% reduction in maximum hematoma thickness. One patient had not yet reached 50% in maximum hematoma thickness, but demonstrated a tendency toward absorption. No cases of postoperative hematoma recurrence were observed during the follow-up period (1–24 months) (Table 2).

**Table 2:** Outcomes of study population.

Variable	Number (*n*%)/mean + SD
Recurrence	0 (0%)
Able to avoid surgery (decreased insize, improved clinically)	25 (100%)
Reduction in size >50%	24 (96%)
Procedure-related complications	2 (8%)
Symptomatic complications	0 (0%)
Embolic material-related complications	0 (0%)
mRS at admission (mRS = 0)	0 (0%)
mRS at last follow-up (mRS = 0)	24 (96%)

SD (standard deviation), SDH (subdural hematoma), mm (millimeters), mRS (modified Rankin Scale score).

Preoperatively, there were 10 cases of mRS score 1 and 15 cases of mRS score 2. At the last follow-up after the interventional procedure, the mRS Score of 1 case was 2, and the rest were 0. The patient with an mRS score of 2 at post-procedural follow-up was a cerebral infarction patient on long-term antiplatelet therapy. The mRS score of 2 was attributed to residual limb weakness from cerebral infarction sequelae, while CSDH-related headache and dizziness symptoms had completely resolved (Table 2).

There were no deaths and 2 asymptomatic complications, including 1 hemorrhage complication and 1 ischemia complication (Table 2). Complications were not related to embolization materials. The patient with a hemorrhagic complication experienced vascular rupture and hemorrhage due to excessive injection pressure by the operator, which was immediately controlled by successful hemostasis achieved through embolization with Embosphere microspheres. One ischemic complication was unrelated to the MMAE procedure itself. It was attributed to the patient’s advanced age, type III aortic arch morphology, and extensive atherosclerotic plaque formation, which led to thrombus formation during pathway establishment, ultimately resulting in middle cerebral artery occlusion.

### Typical case 1

#### Patient

**Figure 1:**
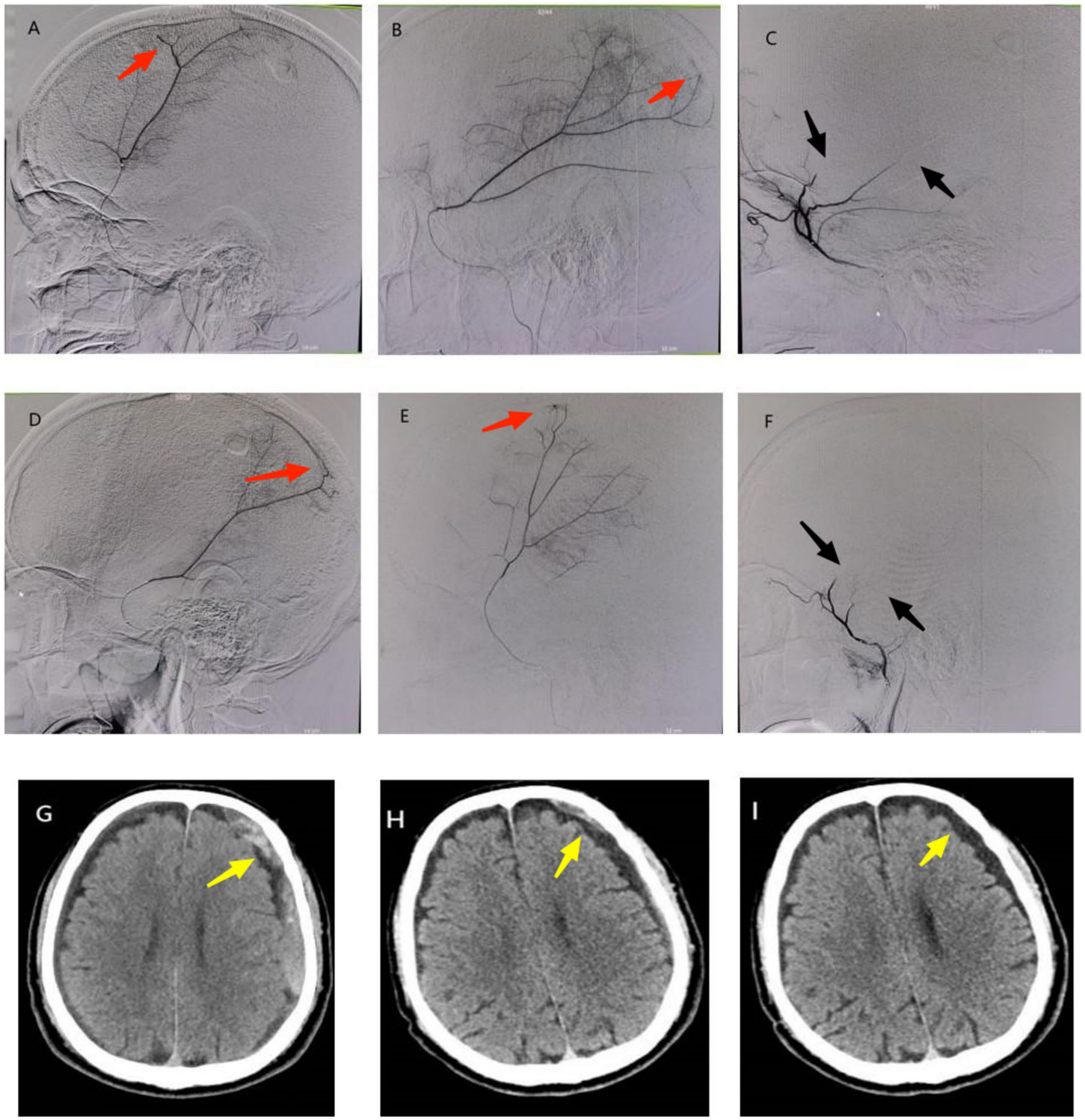
A 70-year-old male patient presented with dizziness and recurrence of left CSDH after burr hole drainage. **(A,B)** Superselective angiography of the right MMA before interventional procedure showed thickening of distal branches and cotton wool-like staining (red arrow); **(D,E)** Superselective angiography of the left MMA before interventional procedure showed thickening of distal branches and cotton wool-like staining (red arrow); **(C,F)** There was no contrast agent filling (black arrow) at the distal end of bilateral MMA branches postoperatively. **(G)** Preoperative cranial CT showed recurrence of left CSDH (yellow arrow). **(H)** Cranial CT at 1 month postoperatively showed obvious absorption of subdural hematoma (yellow arrow). **(I)** Cranial CT at 4 months postoperatively showed subdural hematoma had disappeared with residual subdural effusion (yellow arrow).

Male, 70 years old, presented with “dizziness for 2 days” and underwent follow-up cranial CT on January 8, 2024, revealing recurrence of a left subdural hematoma (Figure 1G). The patient had a documented history of traumatic brain injury 2 months prior and underwent intracranial hematoma subdural burr hole drainage in the neurosurgery department of our hospital on December 14, 2023.

#### Procedure

Under local anesthesia, superselective angiography of the MMA branches demonstrated bilateral distal MMA branch hypertrophy with multifocal cotton wool-like staining (Figures 1A,B,D,E). Subsequent embolization of bilateral distal MMA branches was performed using 100–300 μm Embosphere microspheres (Figures 1C,F). Post-embolization imaging confirmed complete occlusion of the distal MMA branches.

#### Follow-up

Cranial CT scans at 1 and 4 months post-procedure revealed complete hematoma resolution (Figures 1H,I). The patient achieved full symptomatic recovery with an mRS score of 0.

### Typical case 2

#### Patient

**Figure 2:**
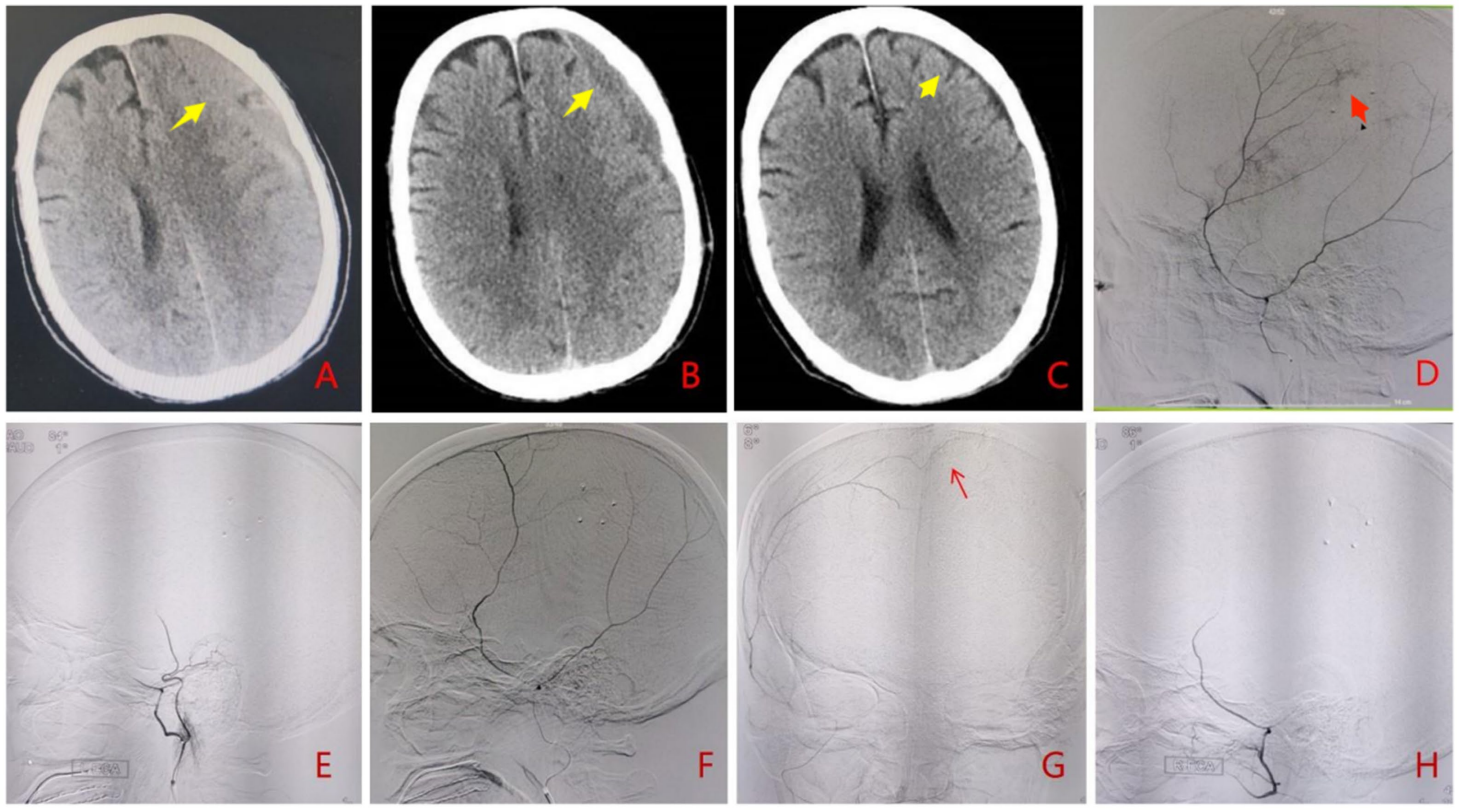
A 73-year-old male patient presented with headache and recurrence of left CSDH after burr hole drainage. **(A)** Preoperative cranial CT showed recurrence of left CSDH (yellow arrow). **(B)** Cranial CT at 1 month postoperatively showed obvious absorption of subdural hematoma (yellow arrow). **(C)** Cranial CT at 4 months postoperatively showed subdural hematoma had disappeared with residual subdural effusion (yellow arrow). **(D)** Superselective angiography of the left MMA before interventional procedure showed thickening of distal branches and cotton wool-like staining (red arrow); **(E,H)** There was no contrast agent filling (black arrow) at the distal end of bilateral MMA branches postoperatively; **(F,G)** Superselective angiography of the right MMA before interventional procedure showed thickening of distal branches and crossing thed midline to form compensatory anastomoses (red arrow).

Male, 73 years old, presented with “Headache for 2 days” and underwent follow-up cranial CT on December 2, 2024, revealing recurrence of a left subdural hematoma (Figure 2A). The patient had no history of trauma and underwent subdural burr hole drainage in the neurosurgery department of our hospital on October 26, 2024.

#### Procedure

Under local anesthesia, selective angiography of the middle meningeal artery (MMA) branches demonstrated hypertrophy of the affected distal vessels, along with multiple areas of cotton-wool-like staining. Additionally, branches from the contralateral middle meningeal artery were observed crossing thed midline to form compensatory anastomoses (Figures 2D,F,G). Subsequent embolization of bilateral distal MMA branches was performed using 100–300 μm Embosphere microspheres (Figures 1E,H). Post-embolization imaging confirmed complete occlusion of the distal MMA branches.

#### Follow-up

Cranial CT scans at 4 months post-procedure revealed complete hematoma resolution (Figures 2B,C). The patient achieved full symptomatic recovery with an mRS score of 0.

## Discussion

Histological studies have confirmed that the formation and progression of CSDH are associated with the development of a vascularized capsule and subsequent leakage within the hematoma. The middle meningeal artery is the blood supply of hematoma capsule (4, 10, 11). Consequently, embolizing the MMA can effectively disrupt the hematoma formation process. In recent years, numerous high-quality studies have demonstrated that MMAE serves as an effective adjunctive therapy to standard treatments (including surgical intervention and medical management) for CSDH, significantly reducing the recurrence rate of this condition (5, 6, 12). Ban’s study (13) has provided new insights into middle meningeal artery (MMA) embolization for chronic subdural hematoma (CSDH). Their comparative study of 541 CSDH patients treated with MMA embolization versus conventional approaches demonstrated markedly lower recurrence rates in both the embolization-only group and the combined treatment group compared to conventional therapy [0% (0/27), 2.2% (1/45), and 27.5% (129/469), respectively]. The STEM study (12) published in the New England Journal of Medicine in 2024, a prospective multicenter randomized controlled trial focusing on symptomatic CSDH, demonstrated that the combined standard therapy (surgical intervention or medical management) with MMAE showed a significantly lower treatment failure rate compared to standard therapy alone (16% vs. 36%, *p* = 0.001). The EMBOLISE trial (5) by Davies JM et al. prospectively enrolled 400 symptomatic CSDH patients, with 197 receiving combined MMAE with surgical evacuation (trial group) and 203 undergoing surgical evacuation alone (control group). The study demonstrated significantly lower recurrence rates in the trial group compared to controls [4.1% (8/197) vs. 11.3% (23/203), *p* = 0.008], indicating that adjunctive MMA embolization reduces recurrence and the rate of surgical retreatment in symptomatic CSDH. These findings substantiate MMAE as an established first-line therapy for symptomatic CSDH patients.

At present, the most commonly used embolization materials for middle meningeal artery embolization include Onyx glue and PVA particles. The curative effect is definite, but there are still some cases of recurrence (14, 15). While Embosphere microspheres have been well-established as an embolic material for various pathologies, including meningiomas, hepatocellular carcinoma, and uterine fibroids, their application in MMAE remains notably underreported in the clinical literature (16–18). Meanwhile, the conclusions obtained from the limited literature reports on Embosphere microspheres are completely opposite (8, 9). Therefore, this study utilized Embosphere microspheres as the embolic material. The results demonstrated a 0% recurrence rate (0/25), no mortality, and absence of Embosphere-related symptomatic complications in the cohort of 25 patients, showing comparable efficacy to contemporary studies in both domestic and international literature (8). Furthermore, all 22 patients who completed cranial CT at 3-month follow-up demonstrated a > 50% reduction in maximum hematoma thickness, consistent with previous reports (19), further validating the efficacy of Embosphere microsphere embolization of MMA for CSDH. The RMPROTECT trial (9) by Shotar E et al. prospectively enrolled 342 CSDH patients who underwent surgery for CSDH recurrence or a first CSDH episode who are at high risk of recurrence, with 171 receiving MMAE (intervention group) and 171 undergoing standard care alone (control group). The 6-month CSDH recurrence rate was 14.8 and 21.0% (*p* = 0.13) in the intervention and control groups, respectively. Its meaning that patients who underwent surgery for CSDH recurrence or a first CSDH episode who are at high risk of recurrence, embolization of the MMA with 300- to 500-μm Embosphere microspheres did not significantly reduce the rate of recurrence at 6 months. This contradicted our research findings that there is no recurrent patient. The Embolization procedure–related complications in this study was slightly higher than the 2.4% reported in RMPROTECT, although all complications observed were asymptomatic and the study had a smaller sample size. The lack of positive results in this study might be directly related to the relatively large diameter of the Embosphere microspheres and Embolization technique. Additionally, patients at high risk of recurrence may often exhibited anastomotic compensation. Thus, we inferred that selecting 100–300 um Embosphere microspheres and performing bilateral MMAE may be reasonable.

Notably, one ischemic complication unrelated to MMAE was attributed to advanced age, type III aortic arch morphology, and extensive atherosclerotic plaque formation. During transfemoral access establishment, intraprocedural thrombus formation led to middle cerebral artery (MCA) occlusion, manifesting as acute-onset motor aphasia and right-sided weakness (Medical Research Council Scale II). Immediate mechanical thrombectomy achieved successful MCA recanalization, with complete neurological recovery and no residual deficits confirmed at 4-month follow-up. Importantly, existing evidence indicates that elderly patients with severe aortic arch tortuosity (Type III) and diffuse atherosclerosis face higher thromboembolic risks during endovascular procedures (20). Recent literature suggests the transradial approach may reduce access-related complications in this population compared to transfemoral routes, representing a feasible alternative for high-risk cases (20). One hemorrhagic complication occurred due to excessive injection pressure during microsphere delivery, resulting in vessel rupture. Immediate embolization with Embosphere microspheres achieved successful hemostasis. Post-procedural imaging confirmed complete occlusion of the bleeding site. The operator emphasized that gentle and meticulous technique is paramount for preventing vascular injury, while highlighting the advantage of Embosphere’s compressibility, allowing precise control in emergent scenarios. This case demonstrates that intraprocedural hemorrhage can be effectively managed through on-the-spot re-embolization without confusion.

Onyx glue is currently the most widely used liquid embolic agent both domestically and internationally. It has the advantages of radiopacity, permanent embolization, and sustainable injection. Disadvantages are vascular toxicity (due to the solvent dimethyl sulfoxide, DMSO), intraoperative pain (necessitating general anesthesia and monitoring), requirement for superselective catheterization to distal target vessels, and high cost (21). The findings regarding different embolic materials remain inconclusive. Some studies have found comparable clinical efficacy between MMAE using different embolic agents [comparing Onyx, n-BCA, and particles] (7). However, Ku J C et al. (15) suggested that Onyx demonstrates superior outcomes in MMAE compared to PVA particles, with CSDH patients exhibiting lower recurrence and retreatment rates. Similar to PVA particles, Embosphere microspheres (22) offer advantages such as non-toxicity, painlessness, suitability for procedures under local anesthesia, and cost-effectiveness. PVA particles (18), with their irregular morphology, tend to aggregate and occlude catheters during delivery. While smaller PVA particles exhibit stronger vascular penetration capability, they often fail to achieve dense embolization of terminal circulation and are more prone to inducing inflammatory reactions. In contrast, Embosphere microspheres (8) are spherical in shape and feature non-absorbability, flow-directed properties, and mechanical embolization capabilities. Available in various sizes, smaller Embosphere microspheres demonstrate enhanced penetration capacity. These microspheres can follow blood flow to distal vasculature, extensively covering terminal branches to achieve permanent, dense, and mechanical embolization of terminal circulation. This effectively limits distal collateral anastomotic compensation and prevents hematoma recurrence. Unlike Onyx, Embosphere microspheres do not require highly superselective catheterization, simplifying the procedure as a single embolization microcatheter suffices. Previous literature (23) reports that the diameter of potentially dangerous anastomotic vessels ranges approximately 50–60 μm. In our center, 100-300 μm Embosphere microspheres are routinely selected for embolization. This approach ensures precise terminal circulation embolization while avoiding occlusion of potentially hazardous anastomotic vascular branches. However, a key limitation is their lack of radiopacity, requiring mixing with contrast agents for fluoroscopic visualization during the procedure.

Bilateral MMA embolization for CSDH: Bilateral MMA branches exhibit cross-midline anastomotic networks, and unilateral embolization may trigger contralateral collateral compensatory blood supply, leading to hematoma recurrence (24, 25). In our cohort, about 2/3 of patients had recurrent or high-risk CSDH. Bilateral MMA embolization using Embosphere microspheres achieved 0% recurrence in these cases, without increasing safety risks. Bilateral MMAE with Embosphere microspheres is strongly advocated to eliminate the compensatory blood supply for recurrent/high-risk CSDH. Emerging evidence is needed to support its application in all chronic subdural hematoma patients as a preventive strategy against recurrence.

## Limitations

1. This study is a single-center retrospective study with a small sample size, so it still needs to be further verified by multi-center and large-sample research.
2. This study is a single-material experience, it can be further compared with traditional treatment methods and other interventional materials.

## Conclusion

Embolization of the middle meningeal artery using Embosphere microspheres is safe and effective in the treatment of chronic subdural hematoma. For patients with recurrence or high risk of recurrence, bilateral middle meningeal artery embolization should be considered.

## Data Availability

The datasets presented in this study can be found in online repositories. The names of the repository/repositories and accession number(s) can be found in the article/supplementary material.
